# ViralPhos: incorporating a recursively statistical method to predict phosphorylation sites on virus proteins

**DOI:** 10.1186/1471-2105-14-S16-S10

**Published:** 2013-10-22

**Authors:** Kai-Yao Huang, Cheng-Tsung Lu, Neil Arvin Bretaña, Tzong-Yi Lee, Tzu-Hao Chang

**Affiliations:** 1 Department of Computer Science and Engineering, Yuan Ze University, Chung-Li 320, Taiwan; 2 Graduate Institute of Biomedical Informatics, Taipei Medical University, Taipei 110, Taiwan

**Keywords:** virus, protein phosphorylation, substrate motif, support vector machine

## Abstract

**Background:**

The phosphorylation of virus proteins by host kinases is linked to viral replication. This leads to an inhibition of normal host-cell functions. Further elucidation of phosphorylation in virus proteins is required in order to aid in drug design and treatment. However, only a few studies have investigated substrate motifs in identifying virus phosphorylation sites. Additionally, existing bioinformatics tool do not consider potential host kinases that may initiate the phosphorylation of a virus protein.

**Results:**

329 experimentally verified phosphorylation fragments on 111 virus proteins were collected from virPTM. These were clustered into subgroups of significantly conserved motifs using a recursively statistical method. Two-layered Support Vector Machines (SVMs) were then applied to train a predictive model for the identified substrate motifs. The SVM models were evaluated using a five-fold cross validation which yields an average accuracy of 0.86 for serine, and 0.81 for threonine. Furthermore, the proposed method is shown to perform at par with three other phosphorylation site prediction tools: PPSP, KinasePhos 2.0 and GPS 2.1.

**Conclusion:**

In this study, we propose a computational method, ViralPhos, which aims to investigate virus substrate site motifs and identify potential phosphorylation sites on virus proteins. We identified informative substrate motifs that matched with several well-studied kinase groups as potential catalytic kinases for virus protein substrates. The identified substrate motifs were further exploited to identify potential virus phosphorylation sites. The proposed method is shown to be capable of predicting virus phosphorylation sites and has been implemented as a web server http://csb.cse.yzu.edu.tw/ViralPhos/.

## Introduction

**Figure 1:**
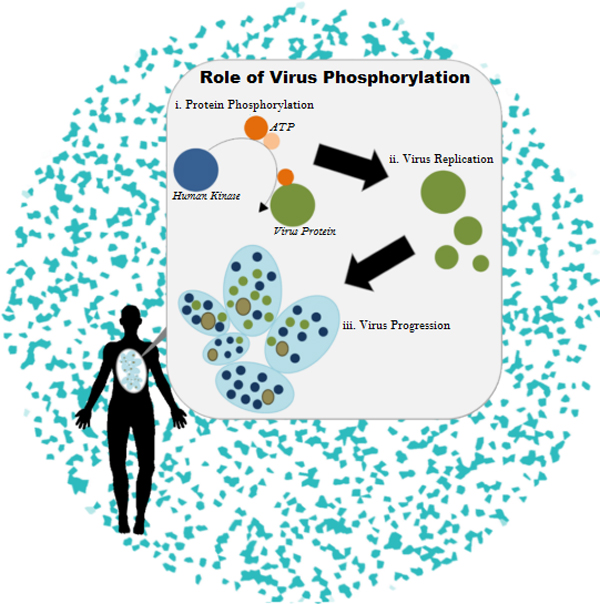
**Conceptual diagram of virus progression**.

A virus is a biological agent capable of interrupting and manipulating normal functions of a cell [1]. In humans, viruses interfere with the normal cellular processes of its host by perturbing the cellular regulatory networks [2]. As shown in Figure 1, viruses undergo phosphorylation by host-cell kinases as a means of enhancing replication and inhibition of normal cellular functions [3]. With the high-throughput of mass spectrometry (MS)-based proteomics [4], an increasing number of virus phosphorylation sites has been identified over the years, including human influenza virus [5], human immunodeficiency virus [6] and the human herpes virus [7].

Protein phosphorylation is a well-studied post-translational modification (PTM) process in eukaryotic cells [4]. The process is initiated by a protein kinase, which transfers of a phosphate group to a target protein substrate - commonly on a serine (S), threonine (T), or tyrosine (Y) residue [8]. Protein substrate sites phosphorylated by a protein kinase agree to a certain linear motif signature. These short linear motifs can be explored in order to further elucidate the interaction between host-cell kinase and virus protein substrates. Also, it will be useful to identify the corresponding kinases that recognize these motifs due to its potential as drug targets [9]. However, previous studies do not consider the corresponding substrate site specificities of catalytic kinases [10].

This study aims to analyze experimentally identified virus phosphorylation sites by bioinformatics analysis. We present a statistical method for identifying potential phosphorylation sites and its potential kinase substrate motifs on virus proteins. In this work, substrate motifs were identified and matched with several well-studied kinase groups as potential catalytic kinases for virus protein substrates. The identified substrate motifs were further exploited to help identify potential virus phosphorylation sites. The method is implemented as a web server, ViralPhos, accessible at http://csb.cse.yzu.edu.tw/ViralPhos/.

## Material and methods

### Data collection and preprocessing

Virus phosphorylation data were collected from major protein databases: virPTM [1], dbPTM [11,12], UniProtKB [13], Phospho.ELM [14]. The virPTM database contains a total of 329 experimentally verified phosphorylation sites on 111 virus protein. Entries from virPTM annotated as "phosphorylated by virus kinases" as well as those not from literature were removed from the collected data resulting to 233, 54, and 14 phosphorylated S (pSer), T (pThr), and Y (pTyr) sites from 104 virus proteins. In dbPTM version 2.0, experimentally verified virus phosphorylation data were obtained and resulted to 51, 15 and 2 phosphorylated S, T and Y sites, respectively, from 32 phosphorylated proteins. Experimentally verified virus phosphorylation data from UniProtKB/Swiss-Prot were also filtered by removing entries annotated as "by similarity", "potential", "probable", and "phosphorylated by virally-encoded kinases" were removed from the original data set resulting to 43, and 12 phosphorylated S, and T sites from 22 virus proteins. From Phospho.ELM version 9.0, experimentally verified virus phosphorylation data were obtained by extracting only entries annotated as "having been identified by using low-throughput processes" resulting to 7, and 2 phosphorylated S, and Y sites from 6 proteins. In order to avoid overlaps, each data obtained from one database is compared to the data obtained from the other databases based on its phosphorylation site position and the UniProtKB accession number utilized by all four databases. Redundancy was removed by retaining only one record in the event of finding multiple records of the same site position and accession number. A summary of the data resources is shown in Additional File 1.

In order to investigate the surrounding residues, with reference to KinasePhos [15,16], sequence fragments were extracted using a window size of 11 centered on S, T, and Y residues. Fragments centered on phosphorylated residues were regarded as positive data while fragments centered on non-phosphorylated residues were regarded as negative data. As shown in Table 1, 233, 54, and 14 positive S, T, and Y fragments as well as 2588, 1170, and 65 S, T, and Y negative fragments were obtained from virPTM. After the removal of redundant fragments among dbPTM, UniProtKB and Phospho.ELM, we have obtained 42, 12, and 2 positive S, T, and Y fragments as well as 352, 106, and 16 negative S, T, and Y fragments for independent testing. In order to avoid a biased prediction performance, the positive data is balanced with the negative data. With reference to previous phosphorylation prediction methods [17-21], a *K*-means clustering method based on sequence identity [22,23] is employed for acquiring a subset that represents the whole negative data set. The number of corresponding positive data is set as the value of *K*, which denotes the number of samples to be obtained from the negative set. This resulted to an equal number of positive and negative S, T, and Y fragments from the data sets as shown in Table 1. Finally, the balanced non-redundant data from virPTM was regarded as the training set while the balanced non-redundant data from dbPTM, UniProtKB and Phospho.ELM were regarded as the independent testing set.

**Table 1:** Data statistics of training set and independent testing set.

	Data set		pSer	pThr	pTyr
**Training set**	virPTM	Positive data	233	54	14
		
		Negative data	2588	1170	65
		
		Balanced negative data	233	54	14

**Independent testing set**	dbPTM	Positive data	42	12	1
		
		Negative data	679	186	11
		
		Balanced negative data	42	12	1
	
	UniProtKB	Positive data	24	10	-
		
		Negative data	217	159	-
		
		Balanced negative data	24	10	-
	
	Phospho.ELM	Positive data	2	-	2
		
		Negative data	67	-	16
		
		Balanced negative data	2	-	2
	
	**Combined non-redundant dataset**	Positive data	42	12	2
		
		Negative data	352	106	16
		
		Balanced negative data	42	12	2

### Motif investigation

**Figure 2:**
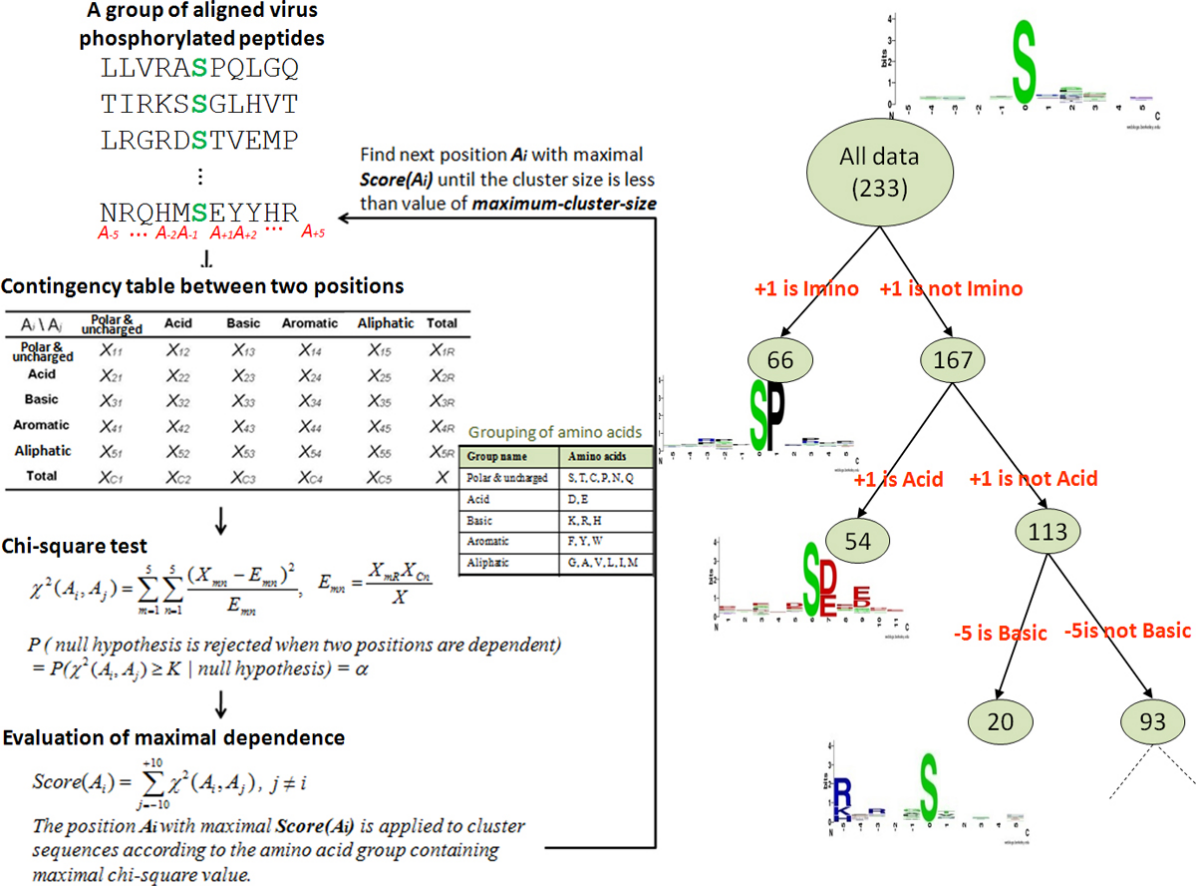
**The analytical flowchart of applying MDDLogo**.

MDDLogo [23] was applied to the training data in order to investigate substrate motif signatures in virus phosphorylation sites. MDDLogo groups a set of aligned sequences to moderate a large group into subgroups that capture the most significant dependencies between positions. Previous works [17,24-26] have proposed the grouping of protein sequences into smaller groups prior to computationally identifying PTM sites. MDDLogo adopts a recursive chi-square test to evaluate the dependence of amino acid occurrence between two positions, A_i _and A_j_, which surround the phosphorylation site. In order to extract motifs that have conserved biochemical property of amino acids, the twenty types of amino acids are categorized into five groups: neutral, acid, basic, aromatic, and imino groups, as shown in Additional File 2. Then, a contingency table of the amino acids occurrence between two positions is constructed, as presented in Figure 2. The chi-square test is defined as:

(1) $$\chi^{2}\left(A_{\text{i}},A_{\text{j}}\right)=\overset{5}{\underset{m=1}{\sum }}\overset{5}{\underset{n=1}{\sum }}\frac{{\left(X_{mn}-E_{mn}\right)}^{2}}{E_{mn}}$$

where *X_mn _*represents the number of sequences that have the amino acids of group m in position A_i _and have the amino acids of group n in position A_j_, for each pair (A_i _, A_j_) with i≠j. *E_mn _*is calculated as $\frac{X_{mR}\cdot X_{Cn}}{X}$, where *X_mR _*= *X_m1_*+ ... +*X_m5_*, *X_Cn _*= *X_1n_*+ ... +*X_5n_*, and *X *denotes the total number of sequences. If a strong dependence is detected (defined as *X^2 ^*that is larger than 34.3, corresponding to a cutoff level of *P *= 0.005 with 16 degrees of freedom) between two positions, then the process is continued as described by Burge and Karlin [27]. As the example shown in Figure 2, position +1 has the maximal dependence with the occurrence of imino amino acids. Subsequently, all data can be divided into two subgroups where one has the occurrence of imino amino acids in position +1 and the other having no occurrence of imino amino acids in position +1. The clustering is a recursive process, which divides the positive set into tree-like subgroups. A parameter, the minimum cluster size, is set when applying MDDLogo to cluster the sequences in the positive set. If the size of a subgroup is less than the given parameter, the subgroup will not be divided any further. In order to obtain an optimal minimum cluster size, MDDLogo is executed using various values. For this study, each subgroup resulting from MDDLogo was represented using WebLogo [28]. These were then visually analyzed to determine if they have conserved motifs.

### Model training and cross-validation

**Figure 3:**
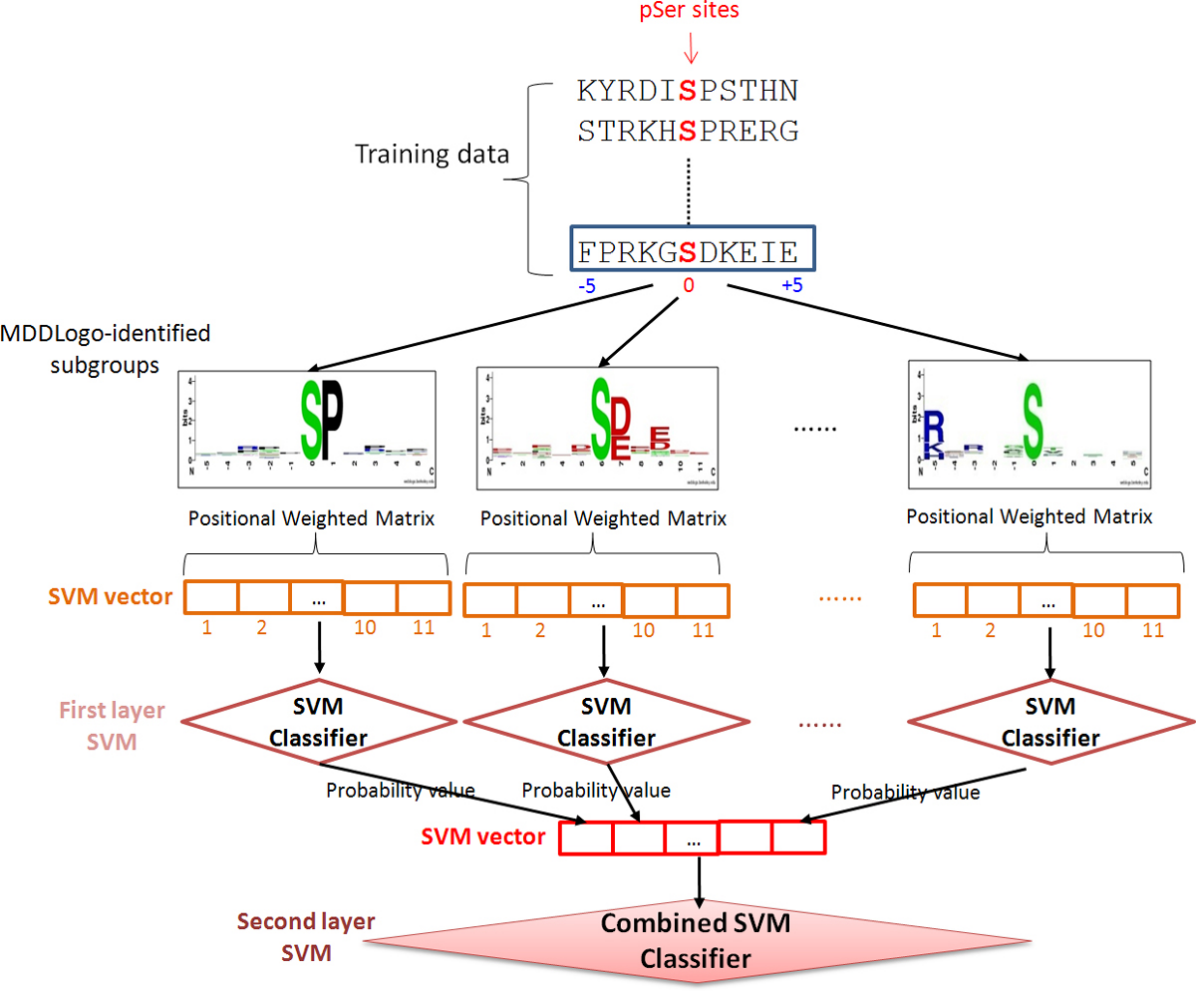
**The conceptual diagram of two-layeredSVMs trained with MDDLogo-identified motifs**.

A five-fold cross-validation evaluation was performed in order to determine which amino acid features were best utilized in establishing models that can effectively identify phosphorylation sites. Support vector machines (SVMs) were generated from the positive data and negative data of the training set. Based on binary classification, the concept behind SVM is to map the input samples into a higher dimensional space using a kernel function, and then to find a hyper-plane that discriminates between the two classes with maximal margin and minimal error. In this work, a public SVM library, LIBSVM [29], was employed to generate the predictive models for each MDDLogo-clustered subgroups. With reference to the encoding method of SulfoSite [30], the positional weighted matrix (PWM), which specifies the relative frequency of amino acids surrounding substrate sites, was utilized in encoding the fragment sequences. A matrix of *m *× *w *elements was used to represent each residue of a training dataset, where *m *stands for the window size and *w *consists of 21 elements including 20 types of amino acids and one for terminal signal. Each MDDLogo-identified substrate motif contained a corresponding PWM with *m *× *w *elements, as illustrated in Figure 3, and a SVM classifier was learned from each PWM. The radial basis function (RBF) $K\left(S_{i},S_{j}\right)=\text{exp}\left(-\gamma ∥S_{i}-S_{j}{∥}^{2}\right)$ was used as the kernel function of the SVMs. The LIBSVM library could output a value of probability estimate ranging from 0 to 1 for each prediction. Thus, the values of probability estimates from each SVM classifier trained with the PWM corresponding to a specific motif were adopted to form an input vector for second-layered SVM.

Prior to the construction of a final model, the predictive performance of models using different parameters were evaluated by performing *k*-fold cross validation. In doing so, the training data was divided into *k *groups by splitting each dataset into *k *approximately equal sized subgroups. During cross-validation, one subgroup is regarded as the test set, and the remaining *k*-1 subgroups are regarded as the training set. The cross-validation process is repeated *k *rounds, with each of the *k *subgroups being used as a test set. The *k *results are then combined to produce a single estimation. The advantage of k-fold cross-validation is that all original data are regarded as both training set and test set, and each data is used for testing exactly once [31]. For this study, *k *was set to five.

The following measures were used to gauge the predictive performance of the trained models: Sensitivity (Sn) = TP/(TP+FN), Specificity (Sp) = TN/(TN+FP), Accuracy [2] = (TP + TN)/(TP+FP+TN+FN), and Matthews Correlation Coefficient $\left(\text{MCC}\right)=\frac{\left(TP\times TN\right)-\left(FN\times FP\right)}{\sqrt{\left(TP+FN\right)\times \left(TN+FP\right)\times \left(TP+FP\right)\times \left(TN+FN\right)}}$, where TP, TN, FP and FN represent the numbers of true positives, true negatives, false positives and false negatives, respectively. After the construction of the predictive model, an independent test was carried out to further evaluate the predictive performance of each SVM. This is done to make sure that the models do not over-fit to the training set [17].

### System integration

The novel method we propose for identifying virus phosphorylation sites with its catalytic kinase was implemented as a web server: http://csb.cse.yzu.edu.tw/ViralPhos/. Data from UniProtKB were integrated into the system in order to allow users to search for virus proteins. Users can query virus protein sequences of interest in order to identify potential phosphorylation sites and its catalytic human kinase. As an output, the system presents virus protein data along with related information including the virus ID, virus name, validated protein interactions collected from VirusMINT [2], and its corresponding literature ID. A sequence comparison tool (BLAST) [32] is also integrated into the system in order to search homologous virus protein sequences for a query sequence.

## Results and discussion

### Substrate motif investigation

Phosphorylated sequences in each MDDLogo-clustered subgroup show a conserved motif representing substrate site specificity. The minimum cluster size was set to 70 for the pSer data, which yielded 6 clusters as shown in Additional File 3. Increasing the minimum cluster size did not result to any clusters, while decreasing the minimum cluster size only resulted to several similar clusters. Based on the entropy plots, it can be observed that some groups contain very similar motifs, some show no conserved motif, and some groups have too little data, which makes the motif unreliable.

For the pThr and pTyr data, the minimum cluster size was set to 20. This resulted to 3 subgroups in pThr and 1 subgroup in pTyr as shown in Additional File 3. However, due to the very low number of pTyr data, the resulting MDDLogo clusters show no conserved motif and contain very few fragments to be considered reliable. Therefore, for this study, pTyr was not further clustered using MDDLogo prior to training a pTyr model. Additionally, to demonstrate the reliability of the MDDLogo clustering method, the MDDLogo-detected motifs were compared with a well-known motif discover tools, Motif-X [33]. Additional File 4 shows potential virus phosphorylation motifs identified by MDDLogo.

### Cross-validation performance

For each model, a threshold parameter was tuned to a specific value that yields a high but balanced specificity and sensitivity result. Table 2 shows the threshold score selected for each model of pSer together with its individual predictive performance and the predictive performance of all MDDLogo-clustered SVM models. MDDLogo clusters exhibiting conserved motifs are shown to be able to yield high predictive accuracies. Specifically, cluster S1, which has a conserved Proline residue at position +1, yields an accuracy of 0.93. On the other hand, MDDLogo clusters that do not seem to have an obvious conserved motif yield a significantly lower predictive performance. For instance, cluster S6, which does not show a strongly conserved motif, only yields an accuracy of a 0.68.

**Table 2:** Five-fold cross validation results on pSer MDDLogo-clustered SVM models.

SVM model	Number of positive data	Number of negative data	Cost value	Gamma value	Sn	Sp	Acc	MCC
All data	233	233	0.5	0.125	0.76	0.72	0.74	0.48

Subgroup S1	66	66	2	0.125	0.98	0.87	0.93	0.86

Subgroup S2	54	54	8	0.03125	0.94	0.92	0.93	0.87

Subgroup S3	34	34	0.5	0.03125	0.91	0.79	0.85	0.71

Subgroup S4	20	20	2	0.125	0.90	0.80	0.85	0.70

Subgroup S5	15	15	2	0.125	0.87	0.80	0.83	0.66

Subgroup S6	44	44	0.5	0.03125	0.75	0.61	0.68	0.37

**Combined performance**					**0.90**	**0.82**	**0.86**	**0.72**

Based on a five-fold cross-validation evaluation, the predictive performance of the MDDLogo-clustered SVMs is significantly better compared to the performance of an SVM model without MDDLogo. As shown in Table 2, the SVM model trained with the combined MDDLogo-clustered motifs yields a higher performance with a sensitivity of 0.90, a specificity of 0.82, an accuracy of 0.86, and a MCC of 0.72 as compared to the SVM with all pSer data which yields a sensitivity of 0.76, a specificity of 0.72, an accuracy of 0.74, and a MCC of 0.48.

Table 3 shows the predictive performance of the pThr models. It can be seen that the pThr SVM model trained with the combined MDDLogo-clustered motifs performs better yielding a sensitivity of 0.83, a specificity of 0.80, an accuracy of 0.81, and an MCC of 0.63 as compared to the SVM model with all pThr data which yields a sensitivity of 0.70, a specificity of 0.70, an accuracy of 0.70, and a MCC of 0.40. Additionally, the cross-validation results on pSer and pThr SVM models trained with unbalanced positive and negative datasets are presented in Additional File 5 and 6, respectively. Due to a lack of virus pTyr data, MDDLogo could not be performed to form SVM model for computationally identifying pTyr sites; thus, a single SVM is used for pTyr until sufficient experimentally verified virus pTyr sites are acquired. The SVM models containing the best predictive performance have been utilized to implement a web-based prediction tool of ViralPhos.

**Table 3:** Five-fold cross validation results on pThr MDDLogo-clustered SVM models.

SVM model	Number of positive data	Number of negative data	Cost value	Gamma value	Sn	Sp	Acc	MCC
All data	54	54	2	0.125	0.70	0.70	0.70	0.40

Subgroup T1	19	19	2	0.125	0.95	0.90	0.92	0.84

Subgroup T2	19	19	2	0.03125	0.95	0.95	0.95	0.89

Subgroup T3	16	16	0.5	0.125	0.68	0.75	0.72	0.44

**Combined performance**					**0.83**	**0.80**	**0.81**	**0.63**

### Independent testing

**Figure 4:**
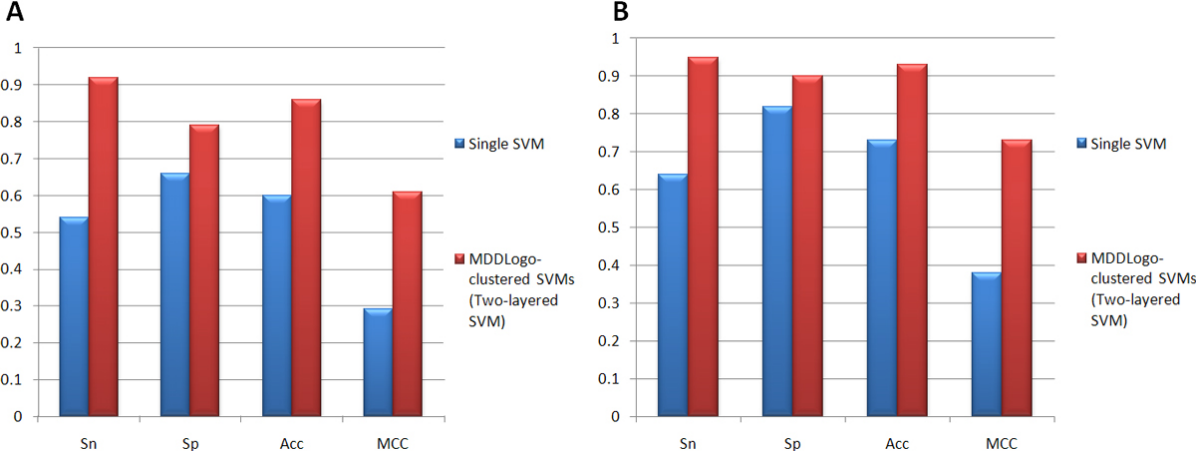
**Comparison of independent testing performance**. (A) Comparison of independent testing results between Single pSer SVM model and MDDLogo-clustered pSer SVM models. (B) Comparison of independent testing results between Single pThr SVM model and MDDLogo-clustered pThr SVM models.

The final non-redundant data set obtained from dbPTM, UniProtKB, and Phospho.ELM consisting of 56 positive sites and 474 negative sites was utilized for further evaluating the MDDLogo-clustered SVMs. As shown in Figure 4A, the SVM model trained using all pSer data yields a sensitivity of 0.54, a specificity of 0.66, an accuracy of 0.60, and the MCC of 0.29. Additionally, using all the pSer MDDLogo-clustered SVMs altogether yields a sensitivity of 0.92, a specificity of 0.79, an accuracy of 0.86, and the MCC of 0.61. On the other hand, Figure 4B shows that using the independent data on Single pThr SVM model yields a sensitivity of 0.64, a specificity of 0.82, an accuracy of 0.73, and the MCC of 0.38. Furthermore, the combined model using all pThr MDDLogo-clustered SVMs was able to yield a sensitivity of 0.95, a specificity of 0.90, an accuracy of 0.93, and the MCC of 0.73.

**Figure 5:**
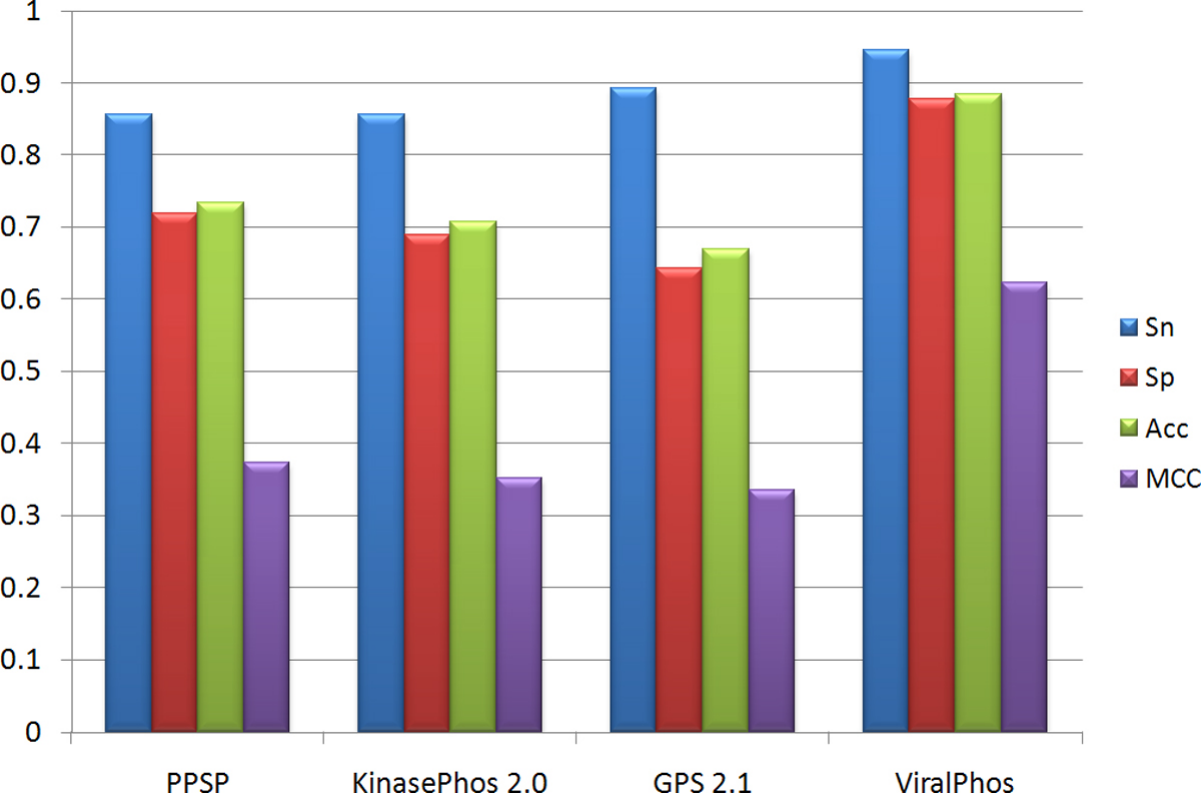
**Comparison of independent testing performance between ViralPhos and other kinase-specific phosphorylation site prediction tools**.

To further demonstrate the effectiveness of the proposed method, the independent testing set is used to compare our method with three popular kinase-specific phosphorylation site prediction tools, PPSP [21], KinasePhos 2.0 [20], and GPS 2.1 [34]. Without any prior information of catalytic kinases for the testing data, all of the kinase-specific models in the prediction tools are chosen for predicting the phosphorylation sites. Figure 5 indicates that all of the prediction tools containing multiple models have a high predictive sensitivity. However, it should be noted that ViralPhos was able to yield a higher specificity compared to the other tools. Since potential kinase information for viral protein phosphorylation sites are still unknown, PPSP yields a higher specificity than KinasePhos and GPS. Overall, the proposed method outperforms the other three tools.

### Motif comparison

In order to identify potential host kinases for virus substrates, the motif of each MDDLogo-generated virus phosphorylation cluster was compared with well-known human kinase substrate motifs from Phospho.ELM. A positional weighted matrix (PWM) was used to represent each MDDLogo-identified substrate motif or Phospho.ELM kinase-specific motif. The measurement of Euclidean distance [35] was applied to calculate the similarity between the PWMs of MDDLogo-identified motif and Phospho.ELM kinase-specific motif. As the scoring calculated by Euclidean distance, the smaller distance value has a higher similarity between two PWMs. Thus, for each MDDLogo-identified motif, the most similar kinase-specific motif is regarded as the matched host kinase and the sequence logo is visualized for further verification.

As shown in Additional File 7, CDK group and MAPK group was found to match with cluster S1 due to a strong similarity with regard to the conserved Proline at position +1. CK2 group was matched with cluster S2 due to a similarly conserved Aspartic acid and Glutamic acid residues at position +3. Furthermore, PKB group was matched with cluster S4 due to a conserved Arginine in position -5 as shown in its respective motifs. In terms of pThr, CDK group and MAPK group were matched with cluster T1 due to a conserved Proline in position +1 as shown in Additional File 8. Cluster T2 was matched to be potentially phosphorylated by CK2 group due to a similarly conserved Aspartic acid and Glutamic acid residues at position +3.

In order to further investigate the identified kinases, a literature survey was done. Reports have been published that CDK group, especially the CDK2, is involved in the transcription and replication of Human Immunodeficiency Virus - 1 by means of phosphorylation [36,37]. Previous studies [10,38] also show that CK2 group phosphorylates Hepatitis C Virus NS5A proteins and Human Immunodeficiency Virus - 1 gp120, gp41, p27, and p17 proteins on both S and T residues. These findings support our MDDLogo-identified groups S2 and T2 matched with CK2 group. With regard to PKB which is matched with cluster S4, it is reported to be involved in the regulation of the Herpes Simplex virus - 1 [39]. Additionally, experimental research also claims that PKB signaling benefits coxsackie virus B3 replication [40].

### Web interface of ViralPhos

**Figure 6:**
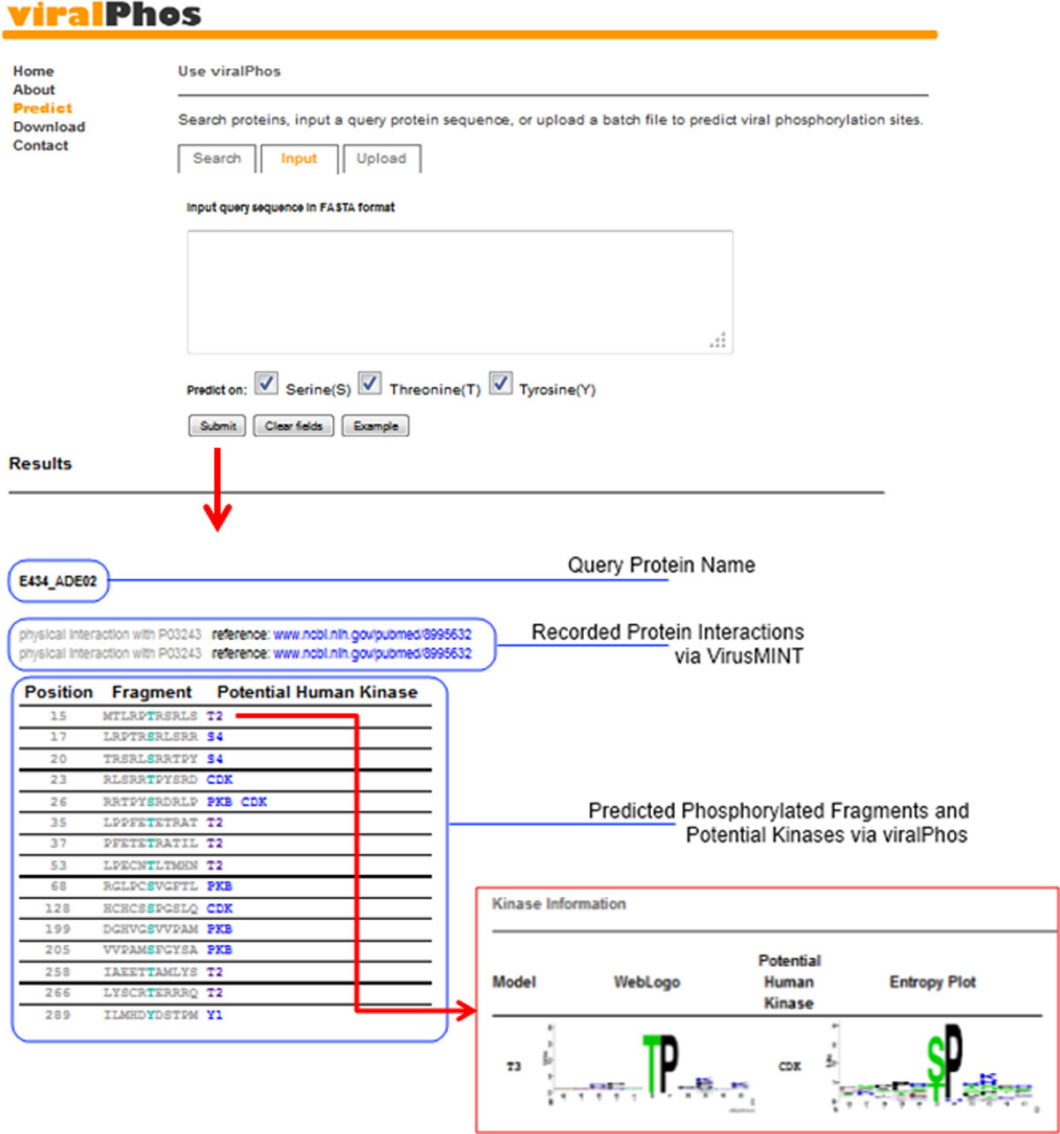
**User interface of ViralPhos**.

To aid in the analysis of virus phosphorylation, ViralPhos has been implemented as a web-based resource freely accessible at http://csb.cse.yzu.edu.tw/ViralPhos/. As shown in Figure 6, users can submit their uncharacterized protein sequences and select the specific residue whose characteristics are to be predicted. The system returns the predictions, including phosphorylated position and flanking amino acids. Users can also access the substrate motifs used for predicting the phosphorylation sites.

## Conclusion

We have developed a novel method for identifying potential virus substrate site specificities and give information on its likely catalytic host kinase. We have identified informative motifs that matched with several well-studied kinase groups including CDK, MAPK, CK2, and PKB as potential catalytic kinases for virus protein substrates. A five-fold cross validation evaluation shows that the proposed method can identify virus phosphorylation sites based on the MDDLogo-identified motifs. Furthermore, an independent test done using data not included in the model training confirms the ability of our MDDLogo-clustered SVMs. The high sensitivity and specificity of MDDLogo-clustered SVMs show that the substrate site motifs are effective for the identification of potential viral protein phosphorylation sites. Overall, this study provides valuable information to the scientific community about what kind of host kinases may be responsible for the phosphorylation of viral proteins. However, it should be noted that the motif result is dependent on the experimentally verified virus phosphorylation sites used as a training data set. Future direction of this work would require the inclusion of a more abundant set of experimentally verified kinase-catalyzed virus phosphorylation sites.

## Competing interests

The authors declare that they have no competing interests.

## Authors' contributions

TYL conceived and supervised the project. KYH, CTL and NAB were responsible for the design, computational analyses, implemented the web-based tool, and drafted the manuscript with revisions provided by TYL and THC. All authors read and approved the final manuscript.

## Availability

ViralPhos can be accessed via a web interface, and is freely available to all interested users at http://csb.cse.yzu.edu.tw/ViralPhos/. All of the data set used in this work is also available for download in the website.

## Supplementary Material

Additional File 1 **Supplementary Table S1**. Data resources of training set and independent testing set Click here for file

Additional File 2 **Supplementary Table S2**. The amino acids group used in MDDLogo clustering Click here for file

Additional File 3 **Supplementary Table S3**. MDDLogo-identified motifs of virus phosphorylation data Click here for file

Additional File 4 **Supplementary Table S4**. Comparison of pSer and pThr motifs between MDDLogo and Motif-X Click here for file

Additional File 5 **Supplementary Table S5**. Five-fold cross validation results on pSer MDDLogo-clustered SVM models trained with unbalanced positive and negative datasets Click here for file

Additional File 6 **Supplementary Table S6**. Five-fold cross validation results on pThr MDDLogo-clustered SVM models trained with unbalanced positive and negative datasets Click here for file

Additional File 7 **Supplementary Table S7**. Motif comparison between MDDLogo-clustered pSer virus motifs and well-studied kinase substrate motifs Click here for file

Additional File 8 **Supplementary Table S8**. Motif comparison between MDDLogo-clustered pThr virus motifs and well-studied kinase substrate motifs Click here for file

## Declarations

The authors sincerely appreciate the National Science Council of the Republic of China for financially supporting publication of this research under Contract Number of NSC 101-2628-E-155-002-MY2.

This article has been published as part of *BMC Bioinformatics *Volume 14 Supplement 16, 2013: Twelfth International Conference on Bioinformatics (InCoB2013): Bioinformatics. The full contents of the supplement are available online at http://www.biomedcentral.com/bmcbioinformatics/supplements/14/S16.
