# Supplementary material for: ViralPhos: incorporating a recursively statistical method to predict phosphorylation sites on virus proteins
Source: BMC Bioinformatics. 2013 Oct 22;14(Suppl 16):S10. doi: 10.1186/1471-2105-14-S16-S10 (PMC3853219; doi:10.1186/1471-2105-14-S16-S10)
Supplement: Additional File 1 — Supplementary Table S1. Data resources of training set and independent testing set [file 1471-2105-14-S16-S10-S1.docx]

**Supplementary Table S1**. **Data resources of training set and independent testing set.**

| **Data set** | **Data resource** |  | **Phosphorylated Proteins** | **pSer** | **pThr** | **pTyr** |
| --- | --- | --- | --- | --- | --- | --- |
| **Training set** | **virPTM** v.1 | Original | 104 | 233 | 54 | 14 |
|  |  | Non-Redundant | 104 | 233 | 54 | 14 |
| **Independent testing set** | **dbPTM 2.0** | Original | 67 | 109 | 33 | 4 |
|  |  | Non-Redundant | 32 | 51 | 15 | 2 |
|  | **UniProtKB**  2012_01_11 | Original | 22 | 43 | 12 | 0 |
|  |  | Non-Redundant | 12 | 24 | 10 | 0 |
|  | **Phospho.ELM**  0910 | Original | 6 | 7 | 0 | 2 |
|  |  | Non-Redundant | 4 | 2 | 0 | 2 |
