# Supplementary material for: ViralPhos: incorporating a recursively statistical method to predict phosphorylation sites on virus proteins
Source: BMC Bioinformatics. 2013 Oct 22;14(Suppl 16):S10. doi: 10.1186/1471-2105-14-S16-S10 (PMC3853219; doi:10.1186/1471-2105-14-S16-S10)
Supplement: Additional File 2 — Supplementary Table S2. The amino acids group used in MDDLogo clustering [file 1471-2105-14-S16-S10-S2.docx]

**Supplementary Table S2.** The amino acids group used in MDDLogo clustering.

| **Group name** | **Amino acids** |
| --- | --- |
| **Neutral** | Threonine (T), valine (V), leucine (L), isoleucine (I), methionine (M), glycine (G), alanine (A), serine (S), cysteine (C) |
| **Acid** | Aspartic acid (D), asparagine (N), glutamic acid (E), glutamine (Q) |
| **Basic** | Lysine (K), arginine (R), histidine (H) |
| **Aromatic** | Phenylalanine (F), tyrosine (Y), tryptophan (W) |
| **Imino** | Proline (P) |
