# Supplementary material for: ViralPhos: incorporating a recursively statistical method to predict phosphorylation sites on virus proteins
Source: BMC Bioinformatics. 2013 Oct 22;14(Suppl 16):S10. doi: 10.1186/1471-2105-14-S16-S10 (PMC3853219; doi:10.1186/1471-2105-14-S16-S10)
Supplement: Additional File 3 — Supplementary Table S3. MDDLogo-identified motifs of virus phosphorylation data [file 1471-2105-14-S16-S10-S3.docx]

**Supplementary Table S3. MDDLogo-identified motifs of virus phosphorylation data.**

| **Residue** | **MDDLogo Cluster** | **Motif** | **Fragments** |
| --- | --- | --- | --- |
| pSer | S1 | 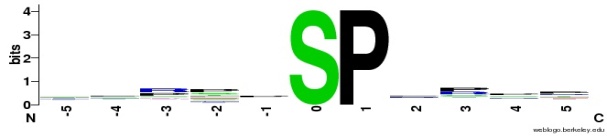 | 66 |
|  | S2 | 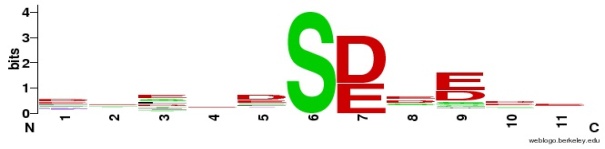 | 54 |
|  | S3 | 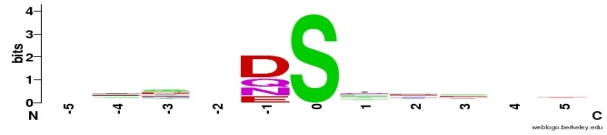 | 34 |
|  | S4 | 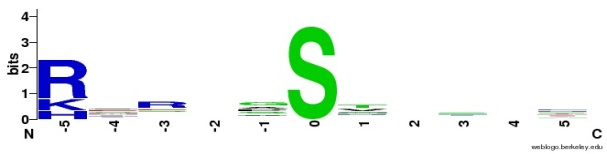 | 20 |
|  | S5 | 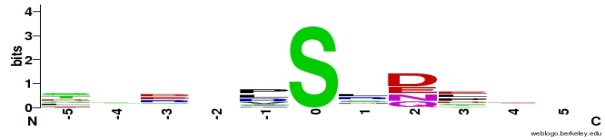 | 15 |
|  | S6 | 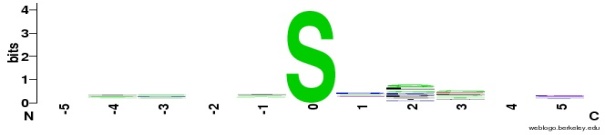 | 44 |
| pThr | T1 | 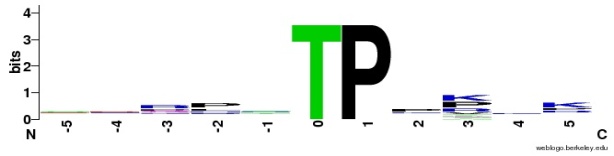 | 19 |
|  | T2 | 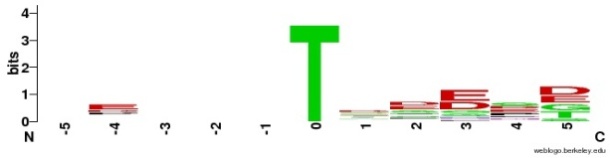 | 19 |
|  | T3 | 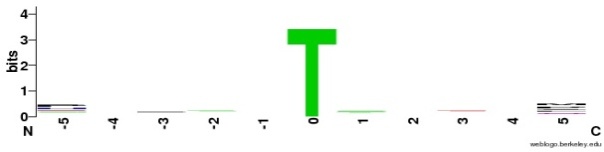 | 16 |
| pTyr | Y1 | 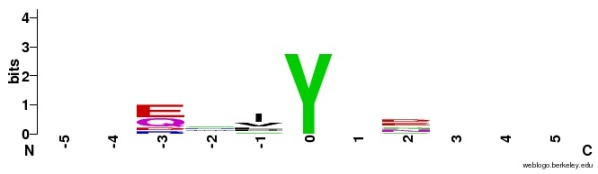 | 9 |
