# Supplementary material for: ViralPhos: incorporating a recursively statistical method to predict phosphorylation sites on virus proteins
Source: BMC Bioinformatics. 2013 Oct 22;14(Suppl 16):S10. doi: 10.1186/1471-2105-14-S16-S10 (PMC3853219; doi:10.1186/1471-2105-14-S16-S10)
Supplement: Additional File 4 — Supplementary Table S4. Comparison of pSer and pThr motifs between MDDLogo and Motif-X [file 1471-2105-14-S16-S10-S4.docx]

**Supplementary Table S4**. Comparison of pSer and pThr motifs between MDDLogo and Motif-X.

|  | **MDDLogo** | | **Motif-X** | |
| --- | --- | --- | --- | --- |
| **Group ID** | **MDDLogo Motif** | **Number of Fragments** | **Motif-X Motif** | **Number of Fragments** |
| **S1** | **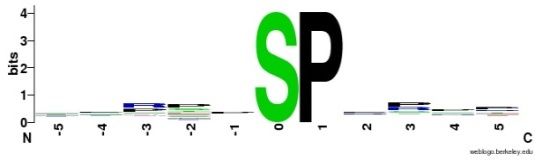** | 66 | **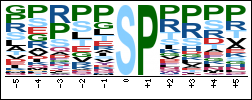** | 66 |
| S2 | 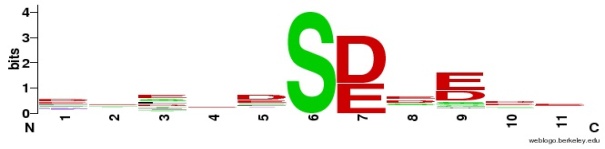 | 54 | 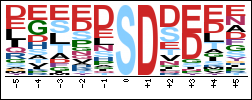 | 33 |
|  |  |  | 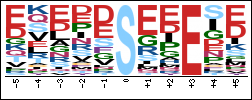 | 26 |
| S3 | 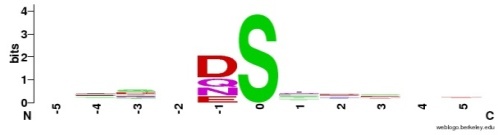 | 34 |  |  |
| S4 | 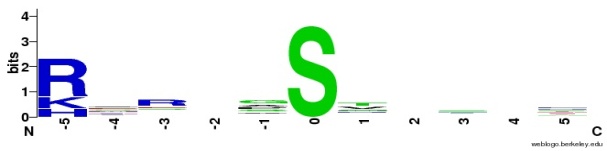 | 20 |  |  |
| S5 | 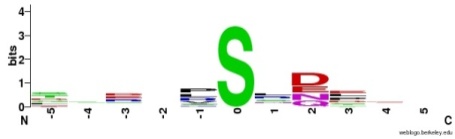 | 15 |  |  |
| S6 | 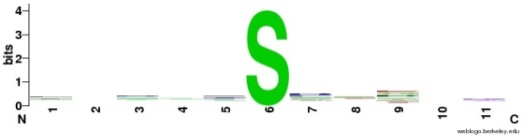 | 44 |  |  |
| T1 | 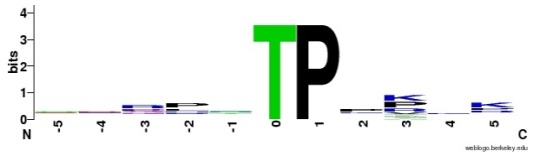 | 19 | 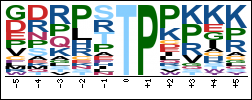 | 19 |
| T2 | 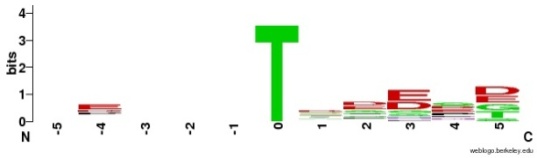 | 19 | 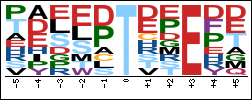 | 10 |
| T3 | 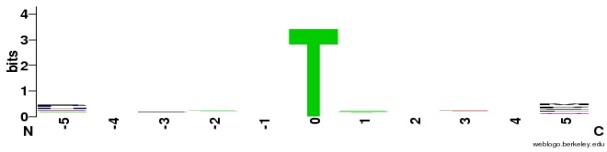 | 16 |  |  |
