# Supplementary material for: ViralPhos: incorporating a recursively statistical method to predict phosphorylation sites on virus proteins
Source: BMC Bioinformatics. 2013 Oct 22;14(Suppl 16):S10. doi: 10.1186/1471-2105-14-S16-S10 (PMC3853219; doi:10.1186/1471-2105-14-S16-S10)
Supplement: Additional File 5 — Supplementary Table S5. Five-fold cross validation results on pSer MDDLogo-clustered SVM models trained with unbalanced positive and negative datasets [file 1471-2105-14-S16-S10-S5.docx]

**Supplementary Table S5. Five-fold cross validation results on pSer MDDLogo-clustered SVM models trained with unbalanced positive and negative datasets.**

| **SVM model** | **Number of positive data** | **Number of non-redundant negative data** | **Cost value** | **Gamma value** | **Sn** | **Sp** | **Acc** | **MCC** |
| --- | --- | --- | --- | --- | --- | --- | --- | --- |
| All data | 233 | 2146 | 0.5 | 0.03125 | 0.74 | 0.70 | 0.70 | 0.28 |
| Subgroup S1 | 66 | 603 | 0.5 | 0.03125 | 0.95 | 0.97 | 0.97 | 0.86 |
| Subgroup S2 | 54 | 482 | 4 | 0.125 | 0.90 | 0.91 | 0.91 | 0.66 |
| Subgroup S3 | 34 | 302 | 0.5 | 0.125 | 0.82 | 0.95 | 0.94 | 0.70 |
| Subgroup S4 | 20 | 182 | 2 | 0.125 | 0.85 | 0.90 | 0.89 | 0.58 |
| Subgroup S5 | 15 | 140 | 8 | 0.125 | 0.73 | 0.87 | 0.86 | 0.46 |
| Subgroup S6 | 44 | 437 | 2 | 0.03125 | 0.61 | 0.88 | 0.86 | 0.39 |
| **Combined performance** |  |  |  |  | **0.80** | **0.93** | **0.92** | **0.63** |
