# Supplementary material for: ViralPhos: incorporating a recursively statistical method to predict phosphorylation sites on virus proteins
Source: BMC Bioinformatics. 2013 Oct 22;14(Suppl 16):S10. doi: 10.1186/1471-2105-14-S16-S10 (PMC3853219; doi:10.1186/1471-2105-14-S16-S10)
Supplement: Additional File 6 — Supplementary Table S6. Five-fold cross validation results on pThr MDDLogo-clustered SVM models trained with unbalanced positive and negative datasets [file 1471-2105-14-S16-S10-S6.docx]

**Supplementary Table S6. Five-fold cross validation results on pThr MDDLogo-clustered SVM models trained with unbalanced positive and negative datasets.**

| **SVM model** | **Number of positive data** | **Number of non-redundant negative data** | **Cost value** | **Gamma value** | **Sn** | **Sp** | **Acc** | **MCC** |
| --- | --- | --- | --- | --- | --- | --- | --- | --- |
| All data | 54 | 1988 | 8 | 0.125 | 0.65 | 0.89 | 0.88 | 0.26 |
| Subgroup T1 | 19 | 699 | 2 | 0.03125 | 0.95 | 0.97 | 0.97 | 0.68 |
| Subgroup T2 | 19 | 699 | 0.5 | 0.03125 | 0.79 | 0.96 | 0.95 | 0.52 |
| Subgroup T3 | 16 | 590 | 0.5 | 0.125 | 0.56 | 0.96 | 0.95 | 0.37 |
| **Combined performance** |  |  |  |  | **0.78** | **0.95** | **0.95** | **0.46** |
