# Supplementary material for: ViralPhos: incorporating a recursively statistical method to predict phosphorylation sites on virus proteins
Source: BMC Bioinformatics. 2013 Oct 22;14(Suppl 16):S10. doi: 10.1186/1471-2105-14-S16-S10 (PMC3853219; doi:10.1186/1471-2105-14-S16-S10)
Supplement: Additional File 8 — Supplementary Table S8. Motif comparison between MDDLogo-clustered pThr virus motifs and well-studied kinase substrate motifs [file 1471-2105-14-S16-S10-S8.docx]

**Supplementary Table S8**. Motif comparison between MDDLogo-clustered pThr virus motifs and well-studied kinase substrate motifs.

| **MDDLogo Cluster** | **Virus Phosphorylation Motif** | **Kinase** | **Substrate Motif of Phospho.ELM** |
| --- | --- | --- | --- |
| **T1** | 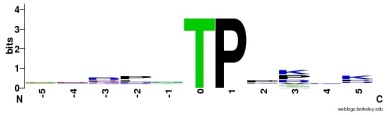 | CDK group | 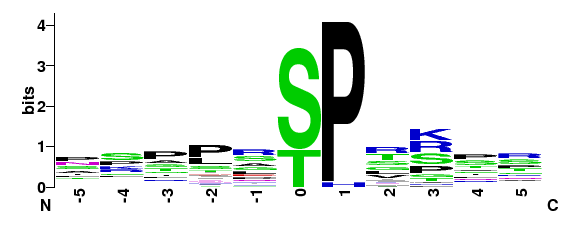 |
|  |  | MAPK group | 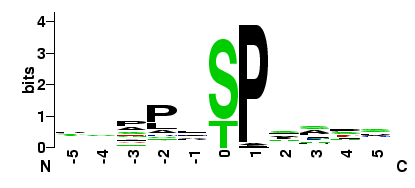 |
| **T2** | 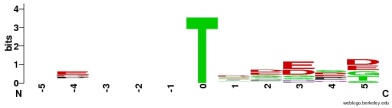 | CK2 group | 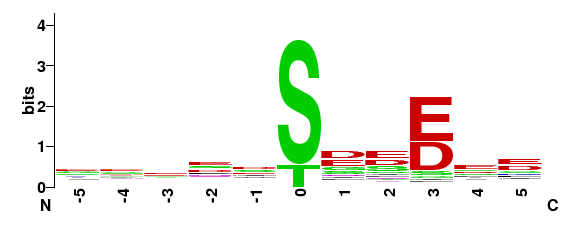 |
